# Mineral Metabolic Abnormalities and Mortality in Dialysis Patients

**DOI:** 10.3390/nu5031002

**Published:** 2013-03-22

**Authors:** Masanori Abe, Kazuyoshi Okada, Masayoshi Soma

**Affiliations:** 1 Division of Nephrology, Hypertension and Endocrinology, Department of Internal Medicine, Nihon University School of Medicine, 30-1, Oyaguchi Kami-chou, Itabashi-ku, Tokyo 173-8610, Japan; E-Mail: kokada@med.nihon-u.ac.jp; 2 Division of General Medicine, Department of Internal Medicine, Nihon University School of Medicine, 30-1, Oyaguchi Kami-chou, Itabashi-ku, Tokyo 173-8610, Japan; E-Mail: souma.masayoshi@nihon-u.ac.jp

**Keywords:** calcium, chronic kidney disease, phosphate, mineral and bone disorder, vascular calcification

## Abstract

The survival rate of dialysis patients, as determined by risk factors such as hypertension, nutritional status, and chronic inflammation, is lower than that of the general population. In addition, disorders of bone mineral metabolism are independently related to mortality and morbidity associated with cardiovascular disease and fracture in dialysis patients. Hyperphosphatemia is an important risk factor of, not only secondary hyperparathyroidism, but also cardiovascular disease. On the other hand, the risk of death reportedly increases with an increase in adjusted serum calcium level, while calcium levels below the recommended target are not associated with a worsened outcome. Thus, the significance of target levels of serum calcium in dialysis patients is debatable. The consensus on determining optimal parathyroid function in dialysis patients, however, is yet to be established. Therefore, the contribution of phosphorus and calcium levels to prognosis is perhaps more significant. Elevated fibroblast growth factor 23 levels have also been shown to be associated with cardiovascular events and death. In this review, we examine the associations between mineral metabolic abnormalities including serum phosphorus, calcium, and parathyroid hormone and mortality in dialysis patients.

## 1. Introduction

Patients with chronic kidney disease (CKD), stage 5D, present with mineral and bone disorder (CKD-MBD) [1,2]. Cardiovascular disease (CVD) is the leading cause of death in dialysis patients, with approximately 50% of cases proving fatal [3,4]. Traditional risk factors for CVD, such as advanced age, hypertension, and smoking, alone cannot fully explain this high prevalence. In addition, disorders of mineral metabolism such as elevated serum calcium, phosphorus, and parathyroid hormone (PTH) levels are associated with increased cardiovascular mortality as well as all-cause mortality [5,6,7,8,9,10,11]. 

A retrospective study of bone mineral metabolism markers in prevalent hemodialysis (HD) patients in Canada found the greatest mortality risk in patients with a combination of high calcium, high phosphorus, and either high or low PTH levels [9]. Independent of phosphorus and PTH levels, increased calcium levels have also been associated with greater all-cause and cardiovascular mortality risk, and poor mental health [5,6,12,13,14]. Moreover, some studies have shown increased mortality in patients with low calcium levels, [15,16] while others failed to do so [5,6]. Markedly increased PTH levels, on the other hand, have been associated with increased mortality, hospitalization, and fractures [5,6,7,12,13,17,18]. 

In an attempt to decrease morbidity and mortality related to CKD-MBD, clinical practice guidelines have been provided in some countries. However, clinically relevant differences exist among these guidelines [19], with survival benefits of calcium, phosphorus, and PTH levels having yet to be confirmed. In this review, we describe the associations between mineral metabolic abnormalities and mortality among dialysis patients, referring to the guidelines of the National Kidney Foundation Kidney Disease Outcome Quality Initiative (KDOQI), Kidney Disease Improving Global Outcomes (KDIGO), and the Japanese Society for Dialysis Therapy (JSDT), as well as other clinical studies (Table 1) [20,21,22,23,24,25,26,27].

**Table 1:** Recommended serum calcium, albumin-corrected calcium, phosphorus, and parathyroid hormone (PTH) levels in patients undergoing dialysis according to different professional organizations, and the lowest mortality risk categories observed in the Dialysis Outcomes and Practice Pattern Study (DOPPS).

Organization	Year	Recommended serum level			
		Calcium (mg/dL)	Albumin-corrected calcium (mg/dL)	Phosphorus (mg/dL)	PTH (pg/mL)
ERA-EDTA [20]	2000	8.8–11.0	-	2.4–4.6	85–170
UK Renal Association [21]	2002	-	8.8–10.4	<5.6	<4× upper normal range
National Kidney Foundation [22]	2003	-	8.4–9.5	3.5–5.5	150–300
Canadian Society of Nephrology [23]	2006	Within normal range	Within normal range	Within normal range	100–500
Australian and New Zealand Society of Nephrology [24]	2006	-	8.4–9.5	2.5–5.5	1–3× upper normal range
DOPPS-derived lowest risk category [25]	2008	8.6–10.0	7.6–9.5 *	3.6–5.0 **	101–600 ***
Japanese Societ for Dialysis Therapy [26]	2008	-	8.4–10.0	3.5–6.0	60–240
KDIGO [27]	2009	-	Within normal range	Within normal range	2–9× upper normal range

ERA-EDTA, European Renal Association-European Dialysis and Transplant Association; DOPPS, Dialysis Outcomes and Practice Patterns Study; JSDT, Japanese Society for Dialysis Therapy; KDIGO, Kidney Disease Improving Global Outcomes; * at 9.6 to 10.0 mg/dL, the risk of mortality increased, but did not achieve statistical significance; ** at 5.1 to 6.0 mg/day, only cardiovascular mortality significantly increased; *** at 100 pg/mL or less and 301 to 600 pg/mL, the risk of mortality increased, but did not achieve statistical significance; - means “not available (N/A)” or “not described”.

## 2. Management of Serum Phosphate Levels

Prolonged hyperphosphatemia causes soft tissue and vascular calcification due, at least in part, to an increase in calcium-phosphate product, and is associated with increased morbidity and mortality [6,7,28,29,30,31,32]. During vascular calcification, hyperphosphatemia exerts a direct calcifying effect on vascular smooth muscle cells [33]. Calcification of coronary arteries, cardiac valves, and pulmonary tissues results in cardiac disease, the leading cause of death in patients with CKD [34,35,36]. Prevention of hyperphosphatemia and serum phosphorus levels maintained within the normal range is therefore imperative.

Several studies have evaluated the relative risk (RR) of mortality associated with serum phosphorus levels in patients treated with HD. Since the target value for phosphorus is based on the results of longitudinal studies, most of which utilize only baseline values, assessment of the influence of laboratory values over time is desirable. Kalantar-Zadeh *et al.* [13] measured serum phosphorus concentrations every three months for a period of two years in 58,058 patients on maintenance dialysis, and revealed through time-dependent Cox models with repeated measures that the serum phosphorus levels associated with the lowest RR of death were between 5.0 and 5.99 mg/dL. In their investigation, the results of the models showed a greater influence of hyperphosphatemia on prognosis than revealed by fixed-covariate Cox models based on baseline values only. Noordzij *et al.* [37] confirmed serum phosphorus levels of 3.5 to 5.5 mg/dL, the control target value of KDOQI, in 1629 incident dialysis patients monitored at six month intervals, and demonstrated that the risk of death decreased when levels remained within this target range. In addition, Noordzij *et al.* [3] also reported significantly higher cardiovascular mortality with phosphorus concentrations above the KDOQI threshold in HD (*n* = 1043) as well as peritoneal dialysis (PD) patients (*n* = 586), using time-dependent Cox models. Furthermore, Block *et al.* [5] reported an association between serum phosphorus concentrations >5.0 mg/dL and an increased RR of death (1.07, 1.25, 1.43, 1.67, and 2.02 for serum phosphorus levels of 5.0 to 6.0, 6.0 to 7.0, 7.0 to 8.0, 8.0 to 9.0, and >9.0 mg/dL) in 40,538 HD patients, while Rodriguez-Benot *et al.* [38] performed a prospective study of 385 patients over 10 years and concluded that mild hyperphosphatemia (5.01–6.5 mg/dL) was an independent risk factor of death in patients on dialysis. In one study in which a reference serum phosphorus range of 4.6 to 5.5 mg/dL was used [9], the relative risk of mortality increased with serum phosphorus levels >6.5 mg/dL, while in another, serum phosphorus levels >6.2 mg/dL were shown to be associated with increased blood pressure, hyperkinetic circulation, increased cardiac work, and high arterial tensile stress [34]. 

Regarding the lower limit of phosphorus, several authors have reported a worsening prognosis at <4 mg/dL or 3 mg/dL [5,9]; however, Nakai *et al.* [39] found no relationship between the three-year survival rate and hypophosphatemia. These results are similar to those of a large-scale study involving 14,829 patients (USRDS) [10]. In another study with a reference range of 5 to 7 mg/dL, the RR of mortality increased with serum phosphorus levels less than or greater than this range [15], and moreover, this increase was particularly significant at >7 mg/dL and <3 mg/dL, respectively. It has also been suggested that serum phosphorus levels <2.5 mg/dL may be associated with abnormalities in bone mineralization such as osteomalacia [40]. Taken together, these findings suggest that the nutritional status of individual patients should be taken into account when evaluating serum phosphate levels in dialysis patients. 

## 3. Dietary Phosphorus Restriction

The use of phosphate-restricted diets, in combination with oral phosphate binders in the management of patients on dialysis, has become well established, having been endorsed by previous guidelines and underlined by appropriate education and counseling to ensure adequate protein intake [1,5]. In most dialysis patients, serum phosphorus concentrations are the result of the balance between net intestinal absorption, net bone resorption, and dialytic removal. The dialytic removal of phosphorus through thrice weekly HD or daily PD is clearly limited and insufficient for maintenance of normal serum phosphorus concentrations in the presence of average intestinal absorption from a typical diet [41,42]. In theory, phosphate binders could prevent hyperphosphatemia even in the presence of a high intake of phosphorus; however, in clinical practice, the effect of current phosphorus binders is limited, particularly when patients are simultaneously exposed to either vitamin D or vitamin D analogue preparations, which is almost always the rule at present [41]. In other words, with the current dialysis prescription and the typically high dietary intake of phosphorus, it is extremely difficult to maintain a normal serum phosphorus concentration even with the use of phosphorus binders. Moreover, simply increasing the dose of phosphorus binders is limited by their own set of side effects. 

Therefore, restriction of dietary phosphorus is a major aspect of patient care in regards to those on dialysis. It is well known that the phosphate supply of a mixed diet is strictly related to the protein content; that is, the dietary intake of phosphorus in a typical diet is close to 14 to 16 mg/g of protein [43]. In addition to dietary phosphate, phosphates are also contained in functional food additives in a wide range of food preparations. This additional phosphorus is not reported in food composition tables, and therefore, does not manifest itself in prescribed dietary schedules; hence it is a so-called hidden phosphorus [41,44]. Cooked ham and roast breast turkey containing phosphate additives were shown to have a phosphorus content 70% higher than samples containing no additives [45,46]. This additive issue is of particular concern because the extra phosphorus is almost completely absorbed by the intestinal tract. These hidden phosphates worsen phosphate balance control and increase the need for phosphate binders, and subsequently, related costs. Sullivan *et al.* [47] conducted a randomized controlled trial (RCT), dividing dialysis patients into two groups: intervention participants (*n* = 145) who received education on how to avoid foods containing phosphorus additives when grocery shopping or visiting fast food restaurants, and control participants (*n* = 134) who continued to receive usual care. The results revealed that education resulted in modest but clinically significant improvements in hyperphosphatemia. The 0.6 mg/dL larger decline in the average phosphorus level among intervention participants, compared with control participants, corresponded to a 5% to 15% reduction in relative mortality risk in observational studies [5,7,9,13,48]. Thus, information and educational programs aimed at dialysis patients are essential in increasing awareness about the existence of phosphate additives in food products.

## 4. Management of Serum Calcium Levels

Since serum calcium levels do not decrease after dialysis, the target range of serum calcium is determined according to (1) the optimal calcium concentration for patient survival, and (2) the normal range of healthy subjects. Reports documenting the association between serum calcium levels and mortality risk in dialysis patients are listed in Table 2. A direct relationship between a higher serum calcium concentration and increased RR of death was observed across the entire spectrum of serum calcium values examined, independent of age, race, sex, diabetes status, vintage, phosphorus, and PTH [5,9]. Furthermore, the risks of higher serum calcium concentrations within narrowly fixed and clinically relevant ranges of serum phosphorus have also been examined, revealing a robust and consistent increase (an approximately 20% increase in RR per mg/dL increase in serum calcium) with increasing serum calcium within each mg/dL stratum of serum phosphorus [5]. In addition, higher serum calcium levels were shown to be consistently associated with an increased risk of death, consistent with findings in studies employing non-time-dependent models [49].

**Table 2:** Association between serum calcium levels and mortality risk in dialysis patients.

Author	Year	Number of subjects	Method of analysis	Reference range of serum calcium (mg/dL)	Inflection range of serum calcium (mg/dL)	HR (95%CI)	*p* value	Outcome
Foley [16]	1996	433	Cox proportional hazards model	>8.8	≤8.8	RR 2.31	0.046	All-cause mortality
Block [9]	1998	2669	Cox proportional hazards model	9.2–9.5	3.7–9.1, 9.6–17.5	N/A	0.12	All-cause mortality
Block [5]	2004	40,538	Cox proportional hazards model	9.0–9.5	>9.5	N/A	<0.05	All-cause mortality
Stevens [8]	2004	515	Cox proportional hazards model	<10.0	10–10.2	RR 1.15 (0.62–2.13)	0.666	All-cause mortality
					10.2–10.6	RR 0.98 (0.52–1.82)	0.94	
					>10.6	RR 1.33 (0.79–2.25)	0.287	
Young [6]	2005	17,236	Cox proportional hazards model	9.0–9.5	>11.4	RR 1.22	<0.05	All-cause and cardiovascularmortality
					<7.8	RR 0.66	<0.0001	
Slinin [10]	2005	14,829	Cox proportional hazards model	≤8.7	>10.2	1.08 (1.01–1.15)	<0.05	Cardiovascular event
				≤8.7	8.8–9.2	1.07 (1.01–1.14)	<0.05	Death
					9.3–9.6	1.05 (0.99–1.12)	NS	
					9.7–10.2	1.11 (1.04–1.18)	<0.05	
					>10.2	1.03 (0.83–1.29)	<0.0001	
Noordzij [37]	2005	1043 (HD)	Time-dependent Cox model	8.4–9.5	<8.3	1.3 (0.7–2.4)	0.40	All-cause mortality
					>9.6	1.0 (0.8–1.4)	0.73	
		586 (PD)	Time-dependent Cox model	8.4–9.5	<8.3	1.4 (0.5–4.2)	0.52	
					>9.6	0.9 (0.6–1.4)	0.63	
Melamed [12]	2006	593	Time-dependent Cox model	8.97–9.33	>9.73	1.52 (1.02–2.26)	<0.05	All-cause mortality
Kalantar-Zadeh [13]	2006	58,058	Time-dependent Cox model	9.0–9.49	>10.5	N/A	< 0.05	All-cause mortality
			Non-time-dependent model	8.0–8.49	>8.5	N/A	<0.05	All-cause mortality
Noordzij [3]	2006	1043 (HD)	Time-dependent Cox model	8.4–9.5	<8.4	1.2 (0.6–2.3)	0.59	CVD-related hospital admission
					>9.5	1.4 (1.1–1.9)	0.01	
		586 (PD)		8.4–9.5	<8.4	4.3 (1.7–10.9)	<0.01	
					>9.5	1.3 (0.8–2.1)	0.35	
		1043 (HD)		8.4–9.5	<8.4	1.5 (0.7–3.4)	0.32	Cardiovascular mortality
					>9.5	1.0 (0.7–1.5)	0.94	
		586 (PD)		8.4–9.5	<8.4	2.8 (0.8–10.1)	0.13	
					>9.5	1.0 (0.5–2.0)	0.98	
		1043 (HD)		8.4–9.5	<8.4	1.1 (0.4–2.7)	0.87	Non-cardiovascular mortality
					>9.5	1.1 (0.8–1.5)	0.69	
		586 (PD)		8.4–9.5	<8.4	0.6 (0.1–4.8)	0.63	
					>9.5	0.8 (0.4–1.5)	0.47	
Kimata [50]	2007	5041	Cox proportional hazards model	8.4–9.0	≥10.4	RR 1.53	<0.05	All-cause mortality
				8.4–9.0	≥10.4	RR 2.29	<0.05	Cardiovascular mortality
Nakai [39]	2008	27,404	Cox proportional hazards model	9.0–9.9	≥10.0	1.098 (1.020–1.182)	0.0129	All-cause mortality
Tentori [25]	2008	25,529	Time-dependent Cox model	8.6–10.0	>10.0	1.16 (1.08–1.25)	<0.0001	All-cause mortality
				8.6–10.0	>10.0	1.24 (1.10–1.41)	<0.05	Cardiovascular mortality
Wald [51]	2008	1846	Cox proportional hazards model	9.1–10.0	>11.0	1.66 (1.09–2.55)	<0.05	All-cause mortality
Miller [52]	2010	107,200	Time-dependent Cox model	9.0–9.4	<9.0	N/A	<0.05	All-cause mortality
					>10.0	N/A	<0.05	
Naves-Diaz [53]	2011	16,178	Time-dependent Cox model	9.5–10.5	10.5–11.0	1.25 (1.02–1.53)	<0.05	All-cause mortality
					>11.0	1.78 (1.40–2.26)	<0.05	
					9.0–9.5	1.25 (1.09–1.44)	<0.05	
					8.5–9.0	1.61 (1.34–1.92)	<0.05	
					<8.5	3.92 (2.95–5.21)	<0.05	
				9.5–10.5	8.5–9.0	1.59 (1.21–2.09)	<0.05	Cardiovascular mortality
					<8.5	3.30 (2.02–5.38)	<0.05	
Noordzij [36]	2011	237		8.4–9.5	<8.4	1.77	0.55	Progression of aortic calcification
					>9.5	3.07 (1.2–8.2)	0.02	

HD, hemodialysis; PD, peritoneal dialysis; RR, relative risk.

In time-dependent models, a higher serum calcium threshold (>10.5 mg/dL) was associated with an increased risk of death, whereas in non-time-dependent models, the mortality predictability of hypercalcemia started at a lower calcium level (>8.5 mg/dL) [13]. A report by Nakai *et al.* [39] on the Patient Registration of the JSDT, revealed a significantly higher mortality risk at three years when baseline serum calcium levels were ≥10.0 mg/dL. In addition, the hazard ratio (HR) for serum calcium levels between 10.0 and 10.9 mg/dL was 1.098 (95% confidence interval 1.020–1.182, *p* = 0.0129) when the reference serum calcium level was set at 9.0–9.9 mg/dL. Furthermore, the NECOSAD study reported that plasma calcium concentrations above the KDOQI threshold increased the RR of CVD-related hospitalization in HD patients [3], while baseline plasma calcium >9.5 mg/dL and iPTH >300 pg/mL were associated with aortic calcification progression, which in turn, was significantly associated with increased risk of all-cause mortality and cardiovascular mortality [36]. 

Kalantar-Zadeh *et al.* [13] reported that hypercalcemia (>8.5 mg/dL in a fixed-covariate model and >10.5 mg/dL in a time-dependent model) continued to remain a strong predictor of an incrementally higher risk of death. In contrast, hypocalcemia was associated with an increased risk of death in unadjusted and case-mix adjusted models (reference calcium group: 9–9.5 mg/dL); however, controlling for malnutrition-inflammation complex syndrome (MICS) surrogates substantially reduced the association between low serum calcium and death [13]. Therefore, the associations traditionally observed between lower serum calcium levels and higher mortality risk [16,54] may be the result of the confounding effect of MICS and its association with the outcome [13]. Miller *et al.* [52] also reported that both low (<9.0 mg/dL) and high (>10.0mg/dL) time-averaged serum calcium levels were associated with increased mortality.

As shown in Table 2, many studies have revealed an association between hypercalcemia and increased all-cause mortality [3,5,6,8,9,10,12,13,16,25,37,39,50,51,52,53]. The inflection point or range at which calcium becomes significantly associated with an increased RR or HR of all-cause mortality varies among studies for the reasons cited above, from >9.5 mg/dL [5] to >9.7 mg/dL [10,12], to >10.0 mg/dL [25,31,52], >10.4 mg/dL [50], >10.5 mg/dL [13,53], >11.0 mg/dL [51], and >11.4 mg/dL [6].

Globally, 50% of dialysis patients have serum calcium levels greater than 9.4 mg/dL and, of these, 25% have serum calcium levels above 10.0 mg/dL [25]. In contrast, at the low end, there is little evidence of an increase in RR until serum levels fall to <8.4 mg/dL [25]. Lowrie and Lew [15] were the first to report increased mortality with calcium levels <9.0 mg/dL in over 12,000 HD patients in 1990. In 1846 prevalent HD patients, Wald *et al.* [51] also reported that serum calcium levels below the KDOQI recommended target of 8.4–9.5 mg/dL were associated with increased mortality. Recently, serum calcium <9.0 mg/dL was shown to increase the HR of mortality in time-dependent multivariable-adjusted analyses [50], while in another study conducted in the United States, the increased RR of mortality with low serum calcium was reversed when findings were adjusted for covariates [5]. On the other hand, in the Dialysis Outcomes and Practice Pattern Study (DOPPS), it was reported that serum calcium levels below 7.8 mg/dL were associated with significantly lower mortality risk [6]. It is therefore unclear at what level of low serum calcium the risk increases. Some clinicians might suggest maintaining a serum calcium level that is as low as possible if within the target range of patients undergoing dialysis. However, it is also important to realize that none of these studies evaluated patients receiving cinacalcet, which lowers calcium through its effects on the calcium-sensing receptor (CaR) while also increasing the receptor’s sensitivity to the cation [27]. Thus, treatment results in an expected decrease in the total serum calcium concentration, and accordingly, further studies are needed to clarify whether such patients are at similar risk to those not on cinacalcet but with identical calcium levels. 

## 5. Assessment of Parathyroid Function

Circulating PTH levels are indicative of parathyroid activity. To assess parathyroid function in CKD patients, an intact-PTH (iPTH) assay system is most frequently applied. It has been reported that high iPTH levels are positively correlated with bone turnover as estimated by bone histomorphometry or bone metabolic markers in CKD patients [55,56,57,58,59]. Therefore, circulating PTH levels are now considered a reliable non-invasive bone turnover marker, as well as being a marker of parathyroid activity; however, alone they are not accurate enough to indicate bone turnover. The gold standard for assessing bone metabolism is bone histomorphometry [60]. 

There is no established consensus on the determination of optimal parathyroid function in dialysis patients. With serum phosphorus and calcium levels, it is widely accepted that standard levels be set based on the relationship with life prognosis [22,26,27]. However, this approach has yet to be applied with circulating PTH levels and, instead, “optimal parathyroid function” is considered “the parathyroid function that would maintain bone turnover similar to that with a normal kidney function” [26]. 

In general, CKD patients require higher parathyroid function than individuals with normal kidney function in order to maintain a similar level of bone turnover. As such, circulating PTH levels approximately two to three times greater than standard levels, as with normal kidney function, are recommended in dialysis patients [26,61]. Some studies have found a “U”-shaped association with increased risk of mortality at both ends [13], although more recent international analyses (DOPPS) have revealed an increased RR of all-cause but not cardiovascular mortality with a PTH >600 pg/mL [25]. The inflection point or range at which PTH becomes significantly associated with increased all-cause mortality varies among studies, ranging from >400 pg/mL [13] to >480 pg/mL [6], >500 pg/mL [15], >511 pg/mL [9], and >600 pg/mL [5]. Unfortunately, however, most of these analyses either do not indicate the assay type or use PTH data measured with multiple assays. Another limiting factor in these studies is that many feature single-baseline PTH values or infrequent (quarterly or less) measurements. On the basis of these observational data, the KDIGO guidelines consider an iPTH level of less than two or greater than nine times the upper limit of normal PTH assay levels to be representative of extreme ranges of risk [27]. Therefore, it is important to recognize that no RCT has yet to show how treatment aimed at achieving specific PTH level results in an improved outcome. In addition, it has not yet been proven that bone turnover, similar to that which occurs with normal kidney function, is beneficial to life prognosis and/or maintenance of daily life activity in dialysis patients [17,54,62,63]. 

Stevens *et al.* [8] assessed various biochemical combinations in concert with dialysis vintage and found that specific risks varied significantly according to three-pronged constellations. The RR for mortality was greatest when levels of serum calcium and phosphorus were elevated in conjunction with low levels of iPTH, and lowest with normal levels of serum calcium and phosphorus in combination with high levels of iPTH. In addition, the duration of dialysis significantly affected the results. A DOPPS study also evaluated combinations of serum parameters of mineral metabolism, but reached slightly different conclusions [25], with elevated serum PTH (>300 pg/mL) and hypercalcemia (>10 mg/dL) being associated with increased mortality risk even under normal serum phosphorus levels. 

Since the contribution of circulating phosphorus and calcium levels on life prognosis seems to be more significant than the effect of parathyroid function [5], it is important that both ions be maintained within standard levels before attempting to control iPTH levels. Further studies aimed at risk-stratifying patients with CKD should also aim to look at combinations of various biochemical abnormalities, rather than isolated parameters. Recommended serum calcium, albumin-corrected calcium, phosphorus, and PTH levels in patients on dialysis, according to different professional organizations, and lowest mortality risk categories of the DOPPS are listed in Table 1 [20,21,22,23,24,25,26,27].

## 6. Medical Treatment for Secondary Hyperparathyroidism

### 6.1. Vitamin D, Calcitriol, and Its Analogs

Active vitamin D deficiency is a common medical condition in patients with CKD [64,65]. Calcitriol, the most active form of vitamin D, increases intestinal calcium absorption, effectively suppresses PTH secretion, and prevents skeletal complications, making it the standard therapy for secondary hyperparathyroidism for more than two decades [66]. It has also been suggested that calcitriol administration may result in elevated serum calcium and phosphorus levels, facilitating vascular calcification and death [67]. Conversely, other studies have shown that the use of calcitriol and other forms of vitamin D derivative is associated with improved survival in patients with cancer or infections [68,69,70]. CKD patient-level outcomes of vitamin D therapy that are considered critical, or of high importance, include mortality, cardiovascular events, rates of hospital admission, parathyroidectomy, fracture, and musculoskeletal pain, and quality of life [27]. 

Although the effects of vitamin D therapy on mortality have not been studied in prospective RCTs, retrospective observational studies suggest that survival of patients on dialysis may be improved by vitamin D therapy [13,71,72,73]. A large historical cohort study demonstrated a significant survival advantage of 20% in HD patients receiving injectable active vitamin D [72]. In addition, a survival benefit of oral active vitamin D was reported in patients receiving mean daily doses of less than 1 µg, with the highest reduction being associated with the lowest dose, compared to patients receiving no oral active vitamin D [74]. Furthermore, a large historical cohort study revealed a survival advantage of the vitamin D_2_ derivative paricalcitol compared with calcitriol [75]. However, in another report on the vitamin D_2_ derivative dexercalciferol, as well as in the DOPPS analyses, this finding was not confirmed (after adjustment for laboratory values and clinical standardized mortality) [73,76]. In addition, the DOPPS revealed no relationship between the use of vitamin D and outcome using an instrumental-variable approach. Therefore, therapy with active vitamin D agents is recommended when parathyroid function greatly exceeds standard levels [77,78]. However, despite this, active vitamin D therapy results in calcemic and often phosphatemic action [79], and thus, more attention should be paid to its safety rather than its efficacy, not least because phosphorus and calcium control is more important than parathyroid control. Supportive therapies, including the application of non-calcium containing oral phosphate binders [80,81], diet, and dialysate containing 2.5 mEq/L [82,83] of calcium may be a safer form of active vitamin D therapy.

### 6.2. Calcimimetics

The traditional treatment for secondary hyperparathyroidism is oral or intravenous administration of vitamin D sterols aimed at lowering PTH levels and (calcium- and non-calcium based) phosphorus binders to control hyperphosphatemia. Although vitamin D sterols have been shown to be effective in suppressing elevated serum PTH levels, they also increase serum phosphorus and calcium levels by stimulating gastrointestinal adsorption. Therefore, only a few patients are able to achieve the recommended therapeutic target [27]. The CaR is a G protein-coupled cell surface receptor that binds calcium ions and senses extracellular levels of calcium ions [84,85]. Calcimimetic agents increase the sensitivity of CaR to extracellular Ca ion levels, leading to decreased PTH synthesis and secretion [86]. In 2004, the US Food and Drug Administration (FDA) approved cinacalcet as the first calcimimetic drug for treatment of secondary hyperparathyroidism. It improves PTH control without increasing circulating levels of calcium and phosphate [87]. Meta-analysis also showed that calcimimetic agents effectively ameliorate iPTH levels in secondary hyperparathyroidism patients and reduce serum calcium and phosphorus disturbances [87]. Moreover, the percentage of patients showing a 30% decrease in serum iPTH levels at the end of treatment was higher in the cinacalcet group than the control group. However, no significant difference was found in all-cause mortality or any adverse events between the calcimimetic and control groups. Further studies are therefore needed to assess the effects of cinacalcet on parathyroid hyperplasia, vascular calcification, bone histomorphometry, and other clinical outcomes in larger samples for longer durations.

## 7. Vascular Calcification

In the general population, atherosclerotic plaque calcification is associated with cardiovascular events such as myocardial infarction, symptomatic angina pectoris, and stroke [88,89,90]. Medial calcification causes arterial stiffness, resulting in elevated pulse pressure and increased pulse wave velocity (PWV), thereby contributing to left ventricular hypertrophy, dysfunction, and failure. Furthermore, it can also result in advanced calcification of the heart and an increased risk of endocarditis. Cardiovascular calcifications are usually progressive, and their extent and severity are highest in patients with CKD [27]. Recent reports also suggest an increased prevalence of cardiovascular calcification in patients in early stages of CKD [28], indicating that a considerable percentage of CKD patients are at high risk of cardiovascular events resulting from vascular calcification. Coronary artery calcification (CAC) is a common and severe problem associated with ischemic cardiovascular disease and mortality in adult dialysis patients [91]. Patients experiencing CAC progression were shown to be at significantly greater risk of experiencing a simultaneous deterioration of markers of arterial compliance and cardiac repolarization [92]. These results suggest that CAC might represent a step in the continuum of events responsible for cardiovascular mortality in patients on dialysis. 

Elevated phosphorus, elevated calcium, oxidized low-density lipoprotein cholesterol, cytokines, and elevated glucose, among others, have been shown to stimulate the transformation of vascular smooth muscle cells into osteoblast-like cells *in vivo* using cell-culture techniques [93]. These factors likely interact at the patient level to increase and/or accelerate calcification in CKD. *In vivo* animal studies have also shown a reduction in arterial calcification with non-calcium-based phosphorus binders compared to calcium-based binders [94,95]. Recently, it has been reported that magnesium prevents phosphate-induced calcification in human aortic vascular smooth muscle cells *in vitro* study [96]. In some studies, risk associations have also been reported between the development and progression of calcification, and epidemiological and biochemical parameters [97,98,99,100]. Age was the most consistent risk factor of severe or progressive calcification, while diabetes, time on dialysis, male sex, high serum iPTH and/or alkalinephosphatase levels, inflammation (*C*-reactive protein levels), calcium intake, hyperphosphatemia, and increased calcium-phosphate product were identified in some studies, but the latter relationship was not uniformly reproduced. 

## 8. Management of Patients with Vascular/Valvular Calcification

Cardiovascular calcification development and progression can be influenced by treatment. Longitudinal studies have also shown that the progression of calcification seems to be modifiable by the choice of phosphate binders. CKD-MBD is a systemic disorder, and therefore, patients with vascular or valvular calcifications should be included in the greatest cardiovascular risk group. It is recommended that the use of calcium-based phosphate binders should be restricted in patients with hypercalcemia, vascular calcification, low levels of PTH, or adynamic bone disease. Accordingly, it should be noted that while treatment with phosphate-binding agents can normalize levels of phosphate and PTH, the use of calcium carbonate favors the progression of vascular calcifications [101]. Moreover, it has been reported that compared with calcium carbonate, sevelamar-HCl provides benefits in all-cause mortality and the composite endpoint of death or dialysis inception, but is not advantageous to dialysis inception in patients with CKD stages three to five and not dependent on dialysis [102]. The cumulative percentage of *de novo* onset of CAC was 12.8% in the sevelamer-treated group and 81.8% in the calcium carbonate-treated group, and in the latter group, the increase in CAC score was also greater [102]. 

Five studies have compared the effects of different phosphate-binder therapies on the progression of CAC scores in chronic HD patients [103,104,105,106,107]. The Treat-to-Goal study (*n* = 200) compared sevelamer-HCl to calcium-containing phosphate binders, analyzing the progression of coronary artery and aortic calcification in prevalent HD patients for one year [103]. Although calcification scores progressed with calcium-containing phosphate binders, treatment with sevelamer-HCl was associated with a lack of calcification progression. A similar design was used, with results showing more calcification progression in patients treated with calcium-based binders compared with sevelamar-HCl in the Renagel in the New Dialysis Patients (RIND) study (*n* = 129), which studied incident HD patients randomized within 90 days after starting dialysis treatment [104]. The median increase in calcification score at 18 months was 11-fold higher in the calcium-treated group compared with the sevelamer-HCl treated group (*p* = 0.01). Block *et al.* [108] assessed all-cause mortality in 127 patients new to HD and assigned to calcium-containing binders or sevelamer-HCl after a median follow-up of 44 months from randomization. The greater risk of death in patients treated with calcium-containing phosphate binders persisted after full multivariable adjustment. In subjects new to HD, baseline CAC score was a significant predictor of all-cause mortality. As a result, they concluded that treatment with sevelamer-HCl was associated with a significant survival benefit compared with calcium-containing phosphate binders [108]. On the other hand, the effect of lanthanum carbonate on progression of vascular calcification, cardiovascular mortality, and all-cause mortality has yet to be systematically studied.

Overall, high calcium intake should be avoided since patients with CKD may encounter difficulties buffering the increased calcium load, and as such, may experience hypercalcemia and/or ectopic calcification [22]. Since calcium overload significantly affects vascular calcification in dialysis patients [28,31], the JSDT guidelines recommend that the dose of oral calcium carbonate not exceed 3.0 g/day [26]. Similarly, the KDIGO guidelines recommend restricting the dose of calcium-based phosphate binders and/or the dose of calcitriol, or vitamin D analog in the presence of persistent or recurrent hypercalcemia during management of hyperphosphatemia [27]. 

Compared with control treatments, no evidence has yet been provided to show that cinacalcet reduces all-cause mortality and cardiovascular mortality. The ADVANCE study [109] evaluated the effects of cinacalcet plus low-dose vitamin D on vascular calcification in HD patients and demonstrated that increases in calcification scores were lower in the aorta, aortic valve, and mitral valve in patients treated with cinacalcet plus low-dose vitamin D sterols. These findings suggest that cinacalcet treatment and low-dose vitamin D sterols may attenuate the progression of established cardiovascular calcification in patients receiving HD. More clinical evidence is now needed to determine whether cinacalcet is associated with a survival benefit in dialysis patients. 

## 9. Fibroblast Growth Factor 23 (FGF23)

FGF23 is a bone-derived hormone that maintains phosphate homeostasis and vitamin D metabolism [110], and increases as renal function declines [111,112,113]. In patients with CKD, elevated FGF23 levels were shown to be associated with left ventricular hypertrophy [114,115] and endothelial dysfunction [116], which are known risk factors of cardiovascular events and death [32,117,118,119]. These results suggest a significant association between FGF23 and CVD in CKD; however, the results related to vascular calcification are conflicting. While several investigators were unable to find a significant relationship between FGF23 and vascular calcification in patients on dialysis and those with early-stage CKD [116,120], others found no association with CAC score in a dialysis setting or with other measures of vascular disease in the general population and in those with reduced estimated glomerular filtration rate [121,122]. Another study of 142 patients with CKD stages two to five, including those on dialysis, found an association between elevated FGF23 levels and higher aortic calcification scores independent of CKD stage and age [123]. 

The identification of elevated FGF23 as a potent risk factor and its potential involvement in adverse outcomes in CKD emphasizes the critical need for therapeutic strategies that lower FGF23. Early physiologic studies performed in healthy volunteers suggest that FGF23 levels may be modifiable through dietary phosphate restriction and phosphate binders [124,125]. Randomized clinical trials are therefore now needed to determine whether FGF23-lowering strategies improve hard clinical endpoints in patients with CKD.

## 10. Conclusions

In patients undergoing dialysis, elevated serum phosphorus is not only associated with secondary hyperparathyroidism and CVD, but also with many other deleterious outcomes, the most important of which is cardiovascular mortality. The association between serum calcium concentration and risk of mortality is generally similar to that of serum phosphorus; however, it is unclear at what level of low serum calcium the risk increases. Since calcium overload significantly affects vascular calcification in dialysis patients, better survival may be achieved by maintaining a serum calcium level that is as low as possible within the standard range of patients on dialysis. Furthermore, the contribution of circulating phosphorus and calcium levels on life prognosis seems to be more significant than the effect of parathyroid function. Thus, serum phosphorus and calcium levels should be maintained within the acceptable normal ranges described in earlier sections before trying to control iPTH levels. At present, there is insufficient evidence to suggest that any specific phosphate binder (calcium- or non-calcium based such as sevelamaer-HCl or lanthanum carbonate) significantly impacts patient-level outcome, and therefore, further studies are needed.
